# Hydrophilic interaction liquid chromatography coupled with tandem mass spectrometry method for quantification of five phospholipid classes in various matrices

**DOI:** 10.1016/j.mex.2023.102026

**Published:** 2023-01-18

**Authors:** Nicolas Mazzella, Aurélie Moreira, Melissa Eon, Arthur Médina, Débora Millan-Navarro, Nicolas Creusot

**Affiliations:** INRAE Nouvelle-Aquitaine Bordeaux, UR EABX, 50 avenue de Verdun, Cestas 33612, France

**Keywords:** Phospholipids, HILIC, HPLC-ESI-MS/MS, Mobile phase pH, Hydrophilic interaction liquid chromatography coupled with tandem mass spectrometry method for quantification of five phospholipid classes in various matrices

## Abstract

Hydrophilic interaction liquid chromatography (HILIC), coupled to tandem mass spectrometry, can be used to separate and determine various polar lipid classes. The development of an HILIC chromatographic separation of several molecular species among five phospholipid classes (PC, PE, PG, PI and PS) is reported here. In this method, a gradient with acetonitrile and 40 mM ammonium acetate buffer was employed. The initial composition was 95% of acetonitrile, then this proportion was decreased to 70% in order to elute all the compounds of interest for a total running time of 11 mins. Furthermore, mobile phase pH can affect the ionizable character of the compounds, according to their pKa values, and also the stationary phase charge state. The influence of such a parameter on both retention times and resolution was evaluated. Besides, the response of different kinds of internal standards (post-extraction standard addition) was evaluated in four different biological matrices, two microalgae extracts and two marine fish extracts. This study found that the recovery rates were between 70 and 140% of the expected value, with relative standard deviations between 10 and 35%, and then limited matrix effects.

• HILIC approach can be used to separate phospholipid according to their polar head-group, and electrospray ionization in negative mode as well as MS/MS allows further identification of the molecular species within each phospholipid class.
• Matrix effects are low and compensated with appropriate internal standards.
• The limits of quantifications were ranging from 0.05 to 0.14 µg.mL^−1^, depending on the analyte.

## Graphical abstract

**Figure:**
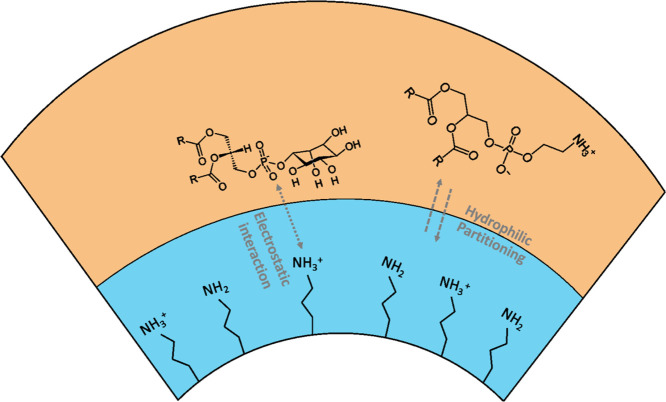

Specifications table

**Table:** 

Subject area:	Chemistry
More specific subject area:	Analytical chemistry
Name of your method:	Hydrophilic interaction liquid chromatography coupled with tandem mass spectrometry method for quantification of five phospholipid classes in various matrices
Name and reference of original method:	Not applicable
Resource availability:	See method details section

**Method details**

## Chemicals and materials

The following polar lipid standards were purchased from Avanti Polar Lipids: 1-palmitoyl-2-oleoyl‑glycero-3-phosphocholine or PC (16:0/18:1) (850,457), 1-palmitoyl-2-oleoyl-sn‑glycero-3-phosphoethanolamine or PE (16:0/18:1) (850,757), 1-palmitoyl-2-oleoyl-sn‑glycero-3-phospho-(1′-rac-glycerol) or PG (16:0/18:1) (840,457), 1,2-diheptadecanoyl-sn‑glycero-3-phosphocholine or PC (2 × 17:0) (850,360), 1,2-diheptadecanoyl-sn‑glycero-3-phosphoethanolamine or PE (2 × 17:0) (830,756), 1,2-dipentadecanoyl-sn‑glycero-3-phosphoethanolamine or PE (2 × 15:0) (850,704), 1,2-diheptadecanoyl-sn‑glycero-3-phospho-(1′-rac-glycerol) or PG (2 × 17:0) (830,456), l-α-phosphatidylserine (Soy, 99%) (sodium salt) (870,336), 1,2-diheptadecanoyl-sn‑glycero-3-phospho-L‑serine (sodium salt) or PS (2 × 17:0) (840,028), and 1-heptadecanoyl-2-(5Z,8Z,11Z,14Z-eicosatetraenoyl)-sn‑glycero-3-phospho-(1′-myo-inositol) (ammonium salt) or PI (17:0_20:4) (LM1502–1EA). l-α-phosphatidylinositol ammonium salt solution, from soybean (79,401–25MG) and ammonium acetate (LiChropur) were provided by Sigma-Aldrich. Acetonitrile, methanol (MeOH) tert‑Butyl methyl ether (MTBE) and isopropanol HPLC grades were purchased from Biosolve Chimie, France. Ultrapure water (UPW) was obtained from Direct-Q® Water Purification System (Merck Millipore).

## Lipid extraction

Biphasic extraction consists of a combination of liquid/solid and liquid/liquid extraction using solvents of different polarities. The aim is to obtain separate hydrophilic phase and a lipophilic phase, this second one should contain lipids of interest [1]. For this purpose, 150 mg of microbeads (0.5 mm diameter) were added in 2 mL microtubes, as well as 10–20 mg (dry mass) of a biological sample. 50 µL of solution of PE (2 × 15:0) at 100 ng.µL^−1^ was added as surrogate, prior to extraction. In this method, the matrices initially extracted were microalgae cultures or fish larvae. These matrices were weighed using a Mettler Toledo NS204S precision balance. First extraction was performed with the addition of 1 mL a MTBE:MeOH (3:1, v/v) mixture, and 650 µL of an UPW:MeOH (3:1, v/v) mixture. The use of the MP Biomedicals FastPrep-24 5 G (3 cycles of 15 s) allowed homogenization of the solution as well as mechanical lysis of the sample via the microbeads, and thus the release of analytes from the sample. A centrifugation (Eppendorf 5430R) at 12 000 RPM allowed to separate the upper lipophilic phase (MTBE) from the lower hydrophilic phase (UPW and MeOH). At this stage, 600 µL of the lipophilic phase was collected. A second extraction (3 cycles of 15 s) was carried out after addition of 700 µL of both MTBE:MeOH (3:1, v/v) and 455 µL of UPW:MeOH (3:1, v/v) mixtures to the residual hydrophilic phase. Once again, after centrifugation, 600 µL of MTBE supernatant was collected and added to the previous one. Only lipohilic organic phases (i.e. 1.1 mL) were kept for further phospholipid analysis. The whole procedure was performed on ice to avoid any enzymatic activities that could lead to a likely alteration of lipid extract. The extracts obtained were stored in a freezer at -80 °C. In addition to the samples, solvent alone (procedural blank) was extracted in order to check the non-contamination of the samples during the extracting step. Prior to HILIC-ESI-MS/MS analysis, 50 µL of internal standards (i.e. PC, PG, PS and PE (2 × 17:0), and PI (17:0_20:4)) at a concentration of 33.3 ng.µL^−1^ were added to the sample. MTBE was evaporated with a gentle stream of N_2_, and then diluted in appropriate volume (typically 250–1000 µL) of isopropanol. The sample can be kept at -18 °C, at least 1 week, until injection.

## HILIC-ESI MS/MS method parameters

Lipid extracts were analyzed with a Dionex Ultimate 3000 HPLC (Thermo Fisher Scientific, France). An API 2000 tandem mass spectrometer (Sciex, France) was used for detection. Chromatographic separation of phospholipids was performed on a Luna NH_2_ HILIC (3 µm, 100 × 2 mm) with a Security Guard cartridge NH_2_ (4 × 2.0 mm). The injection volume and temperature column were set to 20 µL and 40 °C, respectively. The chromatographic separation conditions were reported in Table 1, a final pH value of 6.8 was retained for the ammonium acetate buffer.

**Table 1:** HILIC gradient, mobile phase composition and flow rate.

Time (min)	40 mM ammonium acetate buffer (%)	Acetonitrile (%)	Flow rate (µL.min^−1^)
0	5	95	400
2	5	95	400
7	30	70	400
10	30	70	400
11	5	95	400
13.7	5	95	400

Quantitation of phosphatidylcholine (PC), phosphatidylethanolamine (PE), phosphatidylglycerol (PG), phosphatidylinositol (PI), and were respectively carried out with: PC (16:0_18:1), PE (16:0_18:1), PG (16:0_18:1), PI (16:0_18:2) (about 83% of phosphatidylinositol ammonium salt solution from soybean) and PS (18:2_18:2) (about 58% of phosphatidylserine sodium salt from soybean). PC (2 × 17:0) was used as internal standard for PC phospholipids, PE (2 × 17:0) as internal standard for PE phospholipids, PG (2 × 17:0) as internal standard for PG phospholipids, PI (17:0_20:4) as internal standard for PI phospholipids and PS (2 × 17:0) as internal standard for PS phospholipids. Additionally, PS (2 × 15:0) was used as surrogate for the whole extracting procedure, with a typical recovery of 102 ± 23% (*n* = 10). The mass spectrometry parameter were reported in Table 2, and Q1>Q3 transitions for each molecular species of PC, PG, PE, PI, and PS are provided as Supplementary Material. A first acquisition period from 0 to 6.8 min (for a mobile phase pH=6.8) was used for PC, PE and PG only. The dwell time for each MRM transition was between 3 and 5 ms, resulting in total acquisition time of 1.631 s. The second period, after 6.8 min, was dedicated to PI and PS, and the dwell time was between 5 and 10 ms per transition in this case, for a total acquisition time of 1.764 s. Pause time between mass ranges was systematically set at 0.1 ms. The whole acquisition was operated in negative ionization mode.

**Table 2:** Mass spectrometry parameters.

Phospholipid class	Curtain gaz	CAD	IonSpray	Temperature	Ion source gaz 1	Ion source gaz 2	Declustering potential	Colision energy
PE	30 psi	3	-4500 V	450 °C	30 psi	60 psi	-50 V	-50 V
PG	30 psi	3	-4500 V	450 °C	30 psi	60 psi	-100 V	-50 V
PC	30 psi	3	-4500 V	450 °C	30 psi	60 psi	-100 V	-50 V
PI	30 psi	3	-4500 V	450 °C	30 psi	60 psi	-60 V	-50 V
PS	30 psi	3	-4500 V	450 °C	30 psi	60 psi	-100 V	-50 V

The linear dynamic range was from the limits of quantification for each phospholipid class (Table 5) to 10 µg.mL^−1^ for PC (16:0_18:1), PE (16:0_18:1), PG (16:0_18:1), PI (16:0_18:2), and PS (18:2_18:2), standards. The levels for the internal standard were about 1 µg.mL^−1^. Two quality controls at 0.2 and 2 µg.mL^−1^ were regularly injected during each analytical batch, as well analytical blanks.

## Phospholipid nomenclature

Polar glycerolipids such as phospholipids are constituted of a glycerol backbone esterified by two fatty acids on the *sn-*1 and *sn-*2 positions. The moiety esterified on the *sn-3* position refers to the polar head group (e.g., *sn*-phospho-3-glycerol for the PG). Each polar head group defines a phospholipid or glycolipid class, and each class can be divided into several molecular species according to the fatty acid composition and distribution. Polar glycerolipids are abbreviated as follows: when the fatty acyl chain structures are resolved but the relative *sn-*1 and *sn-*2 positions remain unclear, then the phospholipids are designated PL (C:n_C:n), with C referring to the sum of the number of carbon atoms and n to the number of double bonds for each fatty acyl chain.

## HILIC-ESI-MS/MS analysis of a phospholipid and pH effect on separation

**Fig. 1:**
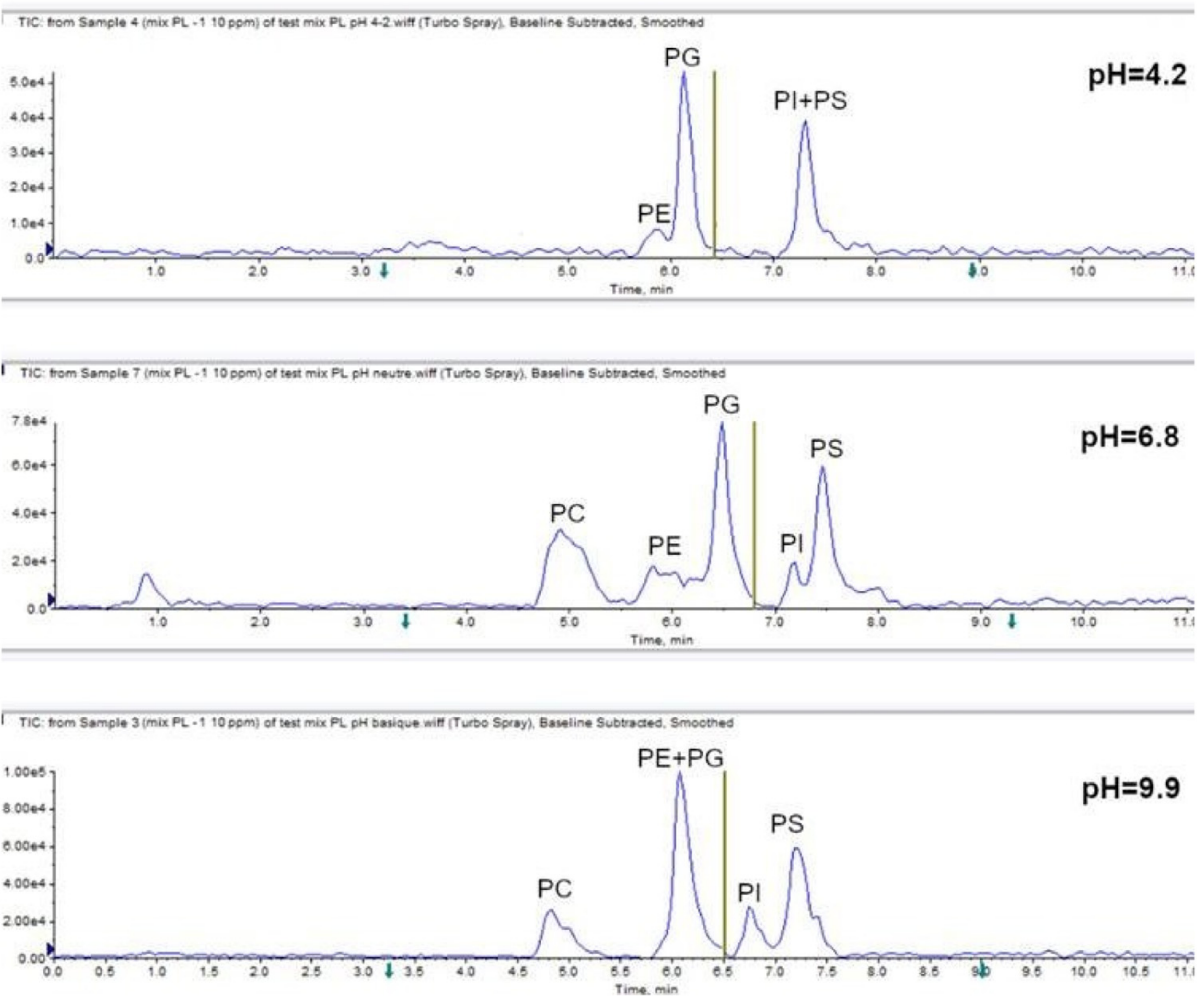
HILIC-ESI-MS/MS analysis of a phospholipid standard mixture, optimization of the separation according to the pH mobile phase. Concentrations ranging from 0.5 to 1 µg.mL^−1^ depending on analyte.

Conventionally, normal-phase liquid chromatograhy (NPLC) and reversed-phase liquid chromatograhy (RPLC) separate lipids based on their polar functional groups and lipophilic composition, respectively [2], [3], [4], [5]. Herein, hydrophilic interaction liquid chromatography (HILIC) was used for the separation and determination of various polar lipid classes. Compared to published RPLC-based approaches, the HILIC separation has the benefit of co-elution of molecular species within each phospholipid class. This typically enables ESI-MS quantification by single standards per class [6]. Regarding this work, a HILIC chromatographic separation of several molecular species among five phospholipid classes (PC, PE, PG, PI and PS) is represented in Fig. 1. Usually, increasing the organic solvent proportion leads to an increase of the retention time. In our case, a gradient with acetonitrile and 40 mM ammonium acetate buffer was employed. The initial composition was 95% of acetonitrile, then this proportion was decreased to 70% in order to elute all the compounds of interest for a total running time of 11 min.

Furthermore, mobile phase pH can affect the ionizable character of the compounds, according to their pKa values [7], and also the stationary phase charge state, especially in the case of an aminopropyl column [8]. For this separation, three pH values were evaluated, ranging from 4.2 (slightly acidic) to 9.9 (slightly basic) adjusted with either acetic acid or ammoniac solution. The retention times, as well as the peak widths and symmetries, are reported in Table 3. The resolutions (between the different phospholipid couples) are shown in Table 4. The most interesting chromatographic conditions were obtained with a neutral pH close to 6.8, for a concentration of 40 mM ammonium acetate into the aqueous buffer. At this pH value, we observed elutions in the following order: PC < PE < PG < PI < PS. PE and PC are in their neutral forms, which justifies their low retention compared to a polar stationary phase. PG and PI have a net negative charge (i.e. deprotoning of the phosphate group), finally PS also has a net negative charge, the amine function of the serine being deprotoned. According to the minimal peak width of 26 s observed for a neutral pH, a total acquisition time around 1.7 s for each of the two periods was sufficient to obtain at least 15 data points across LC peaks. Under acidic conditions, a co-elution of PI and PS was observed (resolution of 0.07) and PC was not detected. For such a zwitterionic compound [9], an adduct with acetate anion (AcO^−^) is necessary to observe a response in electrospray negative ionization mode. Thus, for a pH of 4.2, lower than the pKa (4.8) of the acetic acid, the acetate ionic form would be a minority one, and [PC+AcO]^−^ adduct should not occur anymore. Lastly, for higher pH value (9.9), the resolution between PI and PS couple was improved (R_s_=1.23), whereas PE and PG were here poorly separated, in comparison with neutral pH conditions.

**Table 3:** Retention times (t_R_) and peak widths (ω) in minutes, symmetries, and intensities for each phospholipid standard, and according to 3 pH levels.

pH 4.2	PC	PE	PG	PI	PS
t_R_	N/A	5.9	6.21	7.31	7.33
ω	N/A	0.88	0.32	0.49	0.38
Symmetry	N/A	3.57	1.30	1.10	1.38
Intensity	N/A	2.30E+05	3.89E+05	8.81E+04	4.19E+05
pH 6.8	PC	PE	PG	PI	PS
t_R_	4.93	5.82	6.49	7.19	7.48
ω	0.95	0.85	0.43	0.43	0.45
Symmetry	2.26	2.15	1.62	1.39	1.50
Intensity	2.34E+05	1.90E+05	7.71E+05	2.03E+05	6.47E+05
pH 9.9	PC	PE	PG	PI	PS
t_R_	4.85	5.94	6.1	6.77	7.23
ω	0.68	0.68	0.49	0.57	0.64
Symmetry	3.53	3.53	2.09	2.00	1.78
Intensity	1.90E+05	9.47E+04	1.00E+06	3.00E+05	6.50E+05

**Table 4:** Comparison of resolution (Rs) for the couple of analyte PE/PG and PI/PS, according to 3 pH levels.

Rs	PE/PG	PI/PS
pH 4.2	0.56	0.07
pH 6.8	1.00	0.95
pH 9.9	0.39	1.23

From the literature, it appears that other separations have been carried out before with normal phases like Diol columns, and it was observed in this case a lower retention of PG, which is eluted before PC, PE, PI and PS (the order of elution being unchanged for these latter) [3]. With similar mobile phases (i.e. chloroform/methanol and low amount of aqueous buffer) and silica normal phase, the elution order for three PL in common was PE < PI < PC [4]. Under acidic conditions and HILIC mode with a silica stationary phase, the elution order was PS < PI < PE < PC and the inverted to our observation in this present work [10].

**Fig. 2:**
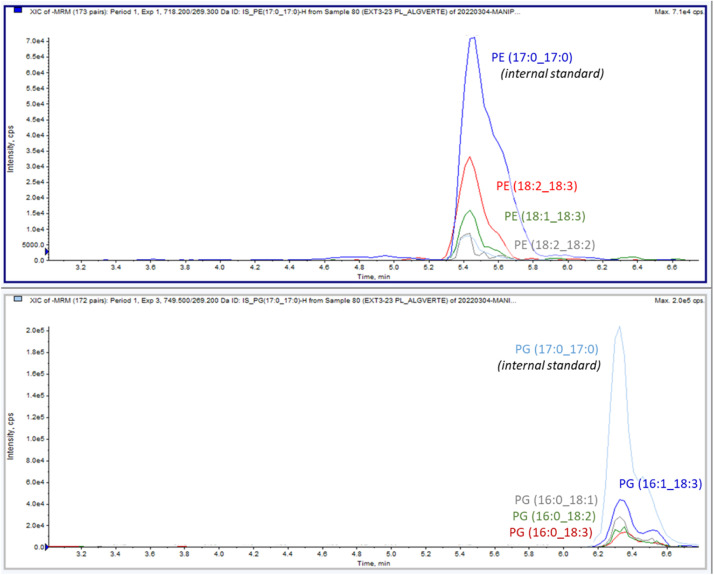
Example of the main molecular species of PE and PG extracted from a green algae culture.

When a co-elution of the molecular species is obtained (i.e. shotgun lipidomics and flow injection analysis, or HILIC separation before MS detection), then quantification accuracy of phospholipid species may be affected by type-II overlap. This kind of overlap is relevant for lipid species differing only by the number of double bonds, mainly because of the natural abundance of ^13^C-atoms. In case of single high resolution mass spectrometry (HRMS), the resolving power become indeed a key parameter [11]. The use of tandem mass spectrometry allows to get rid of this difficulty since we are able to obtain more specific MRM (multiple reaction monitoring) transitions regarding the nature of the acyl chains. It is however to be underlined the possibility of cross-talk according to the resolution applied to the first quadrupole, with regard to a weak mass difference like the presence or not of a double bond, and the selection of the same daughter ion. For example, by selecting the ion *m/z* = 255 associated to 16:0 fatty acyl-chain for the molecular species of PC(16:0_18:0)+AcO (*m/z* = 820) and PC(16:0_18:1)+AcO (*m/z* = 818), there is a likely risk of double detection or false positive when only one compound occur. This difficulty does not appear when the daughter ions are different and when we have a unitary resolution at the third quadrupole. Subsequently, the MRM transitions allow here the identification of the molecular species within each peak corresponding to each phospholipid class (Fig. 2). The response area being proportional to the relative abundance of each molecular species, it is then possible to quantify them individually.

## Evaluation of possible matrix effects and limits of quantification

**Fig. 3:**
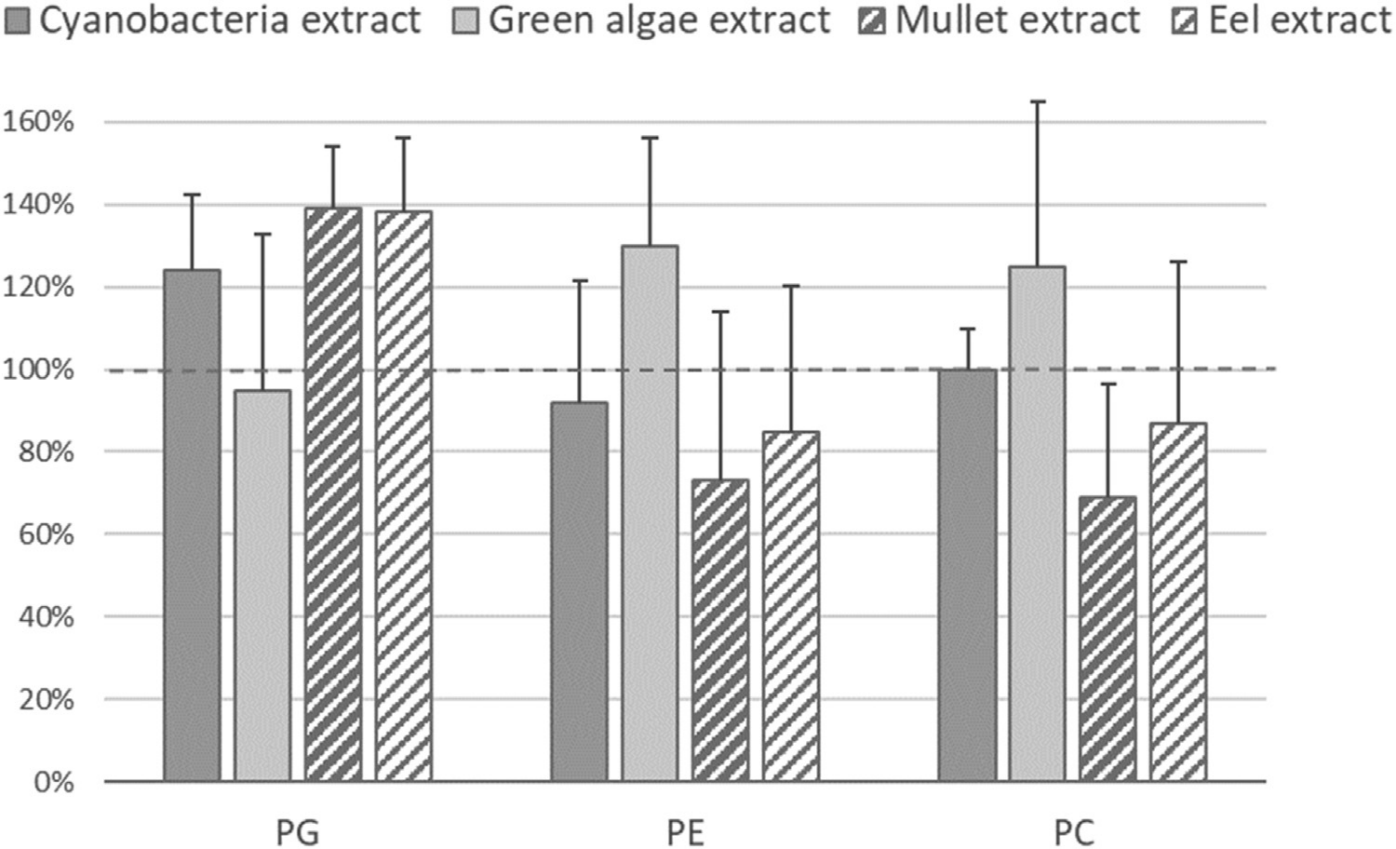
Internal standard recoveries in four different matrices from either microalgae or fishes (*n* = 50 for each matrix and standard).

The response of PG, PE and PC internal standards (post-extraction standard addition) was evaluated in four different biological matrices, two microalgae extracts (*Scenedesmus costatus* and *Phormidium* sp.) and two marine fish extracts (*Chelon auratus* and *Anguilla anguilla*) (Fig. 3). An estimation of recovery rates shows negligible bias, oscillating between 70 and 140% of the expected value, and an acceptable data dispersion, with relative standard deviations between 10 and 35%.

Limits of quantifications were ranging from 0.05 to 0.14 µg.mL^−1^ (Table 5), depending on analyte. These estimates were obtained with the lowest spiking of blank matrix to achieve a signal-to-noise ratio ≥ 10 (*n* = 5 injections). The RSD at this level of concentration was typically < 30%.

**Table 5:** Limits of quantification (LoQ) estimates.

Phospholipid class	LoQ (µg.mL^−1^)
PE	0.14
PG	0.04
PC	0.05
PI	0.2
PS	0.05

## Declaration of Competing Interest

The authors declare that they have no known competing financial interests or personal relationships that could have appeared to influence the work reported in this paper. The authors declare the following financial interests/personal relationships which may be considered as potential competing interests.

## Data Availability

Data will be made available on request.

## Appendix Supplementary Materials

Supplementary Materials Image, application 1
